# Retraction: Antimicrobial, antioxidant and cytotoxic properties of *Chenopodium glaucum *L

**DOI:** 10.1371/journal.pone.0272181

**Published:** 2022-08-03

**Authors:** The PLOS ONE Editors

The PLOS ONE Editors retract this article [1] because it was identified as one of a series of submissions for which we have concerns about authorship, competing interests, and peer review. We regret that the issues were not addressed prior to the article’s publication.

SUK, FU, SF, ESD, SS, SD, and RD did not agree with the retraction. AAR responded but expressed neither agreement nor disagreement with the editorial decision. SM and FA either did not respond directly or could not be reached.
